# In Vitro Comparison of Several Thrombus Removal Tools

**DOI:** 10.3390/jcdd10020069

**Published:** 2023-02-06

**Authors:** Katarzyna Pigoń, Natalia Tomecka, Dominika Korner, Maciej Pękała, Sławomir Grzegorczyn, Adam Konka, Ewa Nowalany-Kozielska, Andrzej Tomasik

**Affiliations:** 1 II Department of Cardiology in Zabrze, Faculty of Medical Sciences in Zabrze, Medical University of Silesia, 40-055 Katowice, Poland; 2 Students’ Scientific Group at II Department of Cardiology, Faculty of Medical Sciences in Zabrze, Medical University of Silesia, 40-055 Katowice, Poland; 3 Kardio-Med Silesia, 41-800 Zabrze, Poland; 4 Department of Biophysics, Faculty of Medical Sciences in Zabrze, Medical University of Silesia, 40-055 Katowice, Poland

**Keywords:** thrombus, aspiration thrombectomy, mechanical thrombectomy, aspiration catheters, stentriever

## Abstract

Background: Although the routine use of thrombus aspiration is not recommended, the thrombectomy technique still might be considered for a selected population of patients. Therefore, the assessment of the effectiveness of commercially available thrombectomy devices is still clinically relevant. Aim: Here, we present an in vitro comparison of several different types of catheters that can be used for thrombus aspiration or removal. Methods: Through the removal of 6 h and 24 h human blood clots in an in vitro model, four catheters were compared: the Launcher, Pronto V4, Vasco+ and the stent-retriever Catchview. The aspiration efficacy was expressed as a percentage of the initial thrombus weight. The effectiveness of the patient’s aspiration was dependent on the time of thrombus formation and was significantly higher for a thrombus formed over 24 h (58.5 ± 26.5%) than for one formed over 6 h (48.0 ± 22.5%; *p* < 0.001). In the presented in vitro model, Pronto V4 and Launcher showed the highest efficiency. Conclusions: Large-bore aspiration catheters were found to be more effective than narrow-bore catheters or stent-retrievers in an in vitro model of thrombus removal. The thrombus aspiration efficacy increases with longer thrombus formation times.

## 1. Introduction

Thrombus formation is the leading cause of acute myocardial infarction. When it can be performed in a timely manner, primary percutaneous coronary intervention (pPCI) is the preferred and most effective reperfusion strategy for patients with STEMI [1]. However, some of the coronary angioplasty limitations can not only diminish the expected benefits of the restoration of myocardial perfusion [2,3] but also increase the mortality rate of patients [4]. Striving to achieve procedural optimization, we recently witnessed the introduction of thrombus aspiration as an adjunct to pPCI, putting an equally quick end to hopes for its widespread clinical application. Due to the publication of the results of the TOTAL and TASTE trials [5,6], which questioned the effectiveness and safety of thrombus aspiration in STEMI patients, its routine use was downgraded to a class III indication [7]. The majority of patients enrolled in these highly influential studies were treated with two types of aspiration catheters. In the TOTAL study, all the aspirations were performed with an Export catheter [8]. Comparably, in the TASTE study, 84% of the patients were treated with either Export or Eliminate catheters, both of which were deemed ineffective in the optical coherence tomography assessment of the reduction in the thrombus burden [9,10]. Moreover, in an in vitro experiment that tested the catheters’ performance in the aspiration of saline or blood clots [11], both catheters scored among the worse half of the entire group of examined catheters.

Thus, the substantial technological question arises as to whether we are using the right tools for thrombus removal. The answer to the question is of particular relevance for the group of patients with a high thrombus burden, among whom the trends toward reduced rates of cardiovascular death and increased rates of stroke or transient ischemic attack have been observed and provide a rationale for future trials of improved thrombus aspiration technologies among this high-risk subgroup [12]. Considering all these data, we aimed to compare the effectiveness of several thrombus aspiration/removal tools in an in vitro experiment.

## 2. Materials and Methods

### 2.1. Materials

The selection of thrombus removal devices for this bench test was based on publications of case reports or case series [13,14,15,16,17,18] and the results of our stratified meta-analysis [19]. We tested the thrombus removal efficacy of three manual aspiration catheters and one stentriever. Their specifications are described in Table 1.

The different diameters of the tested catheters were intended to reflect their deliverability to all segments of the anatomically tapering coronary vasculature. The manual thrombectomy devices are simpler to use than their motorized counterparts. Most of them are composed of monorail catheters with a central lumen, which is connected through one or more holes located at the tip. Manual suction is performed with a syringe. The Pronto V4 (Vascular Solutions, Minneapolis, MN, USA), Launcher (Medtronic, Minneapolis, MN, USA), CatchView (Balt, Montmercy, France) and Vasco+ (Balt) catheters were investigated in this study. According to the instructions, CatchView is used for flow restoration in patients with ischemic stroke caused by large intracranial vessel occlusion. It is unique in providing the possibility of the compression of the stentriever in the small vessels and its ability to expand by up to 6 mm in diameter. Vasco+ is a reinforced micro-catheter intended for the injection of diagnostic and therapeutic products or use with the self-expanding stents LEO+ or SILK+. It was designed to provide access and support in the treatment of intracranial aneurysms and mechanical thrombectomy. The Pronto V4 extraction catheter is intended for the removal of emboli or thrombi from the vessels in the coronary and peripheral vasculature. To quote the manufacturer, Pronto V4 has a uniformly large extraction lumen and patented self-centered Silva Tip. Launcher is one of the most commonly used coronary guide catheters. In a study by Hara et al., which included in vitro models for the comparison of the catheters’ efficacy, only aspiration catheters were compared [11]. In a study by Rioufol et al., a different guide catheter (Cordis, Santa Clara, CA, USA) was tested [20].

### 2.2. Methods

**Figure 1:**
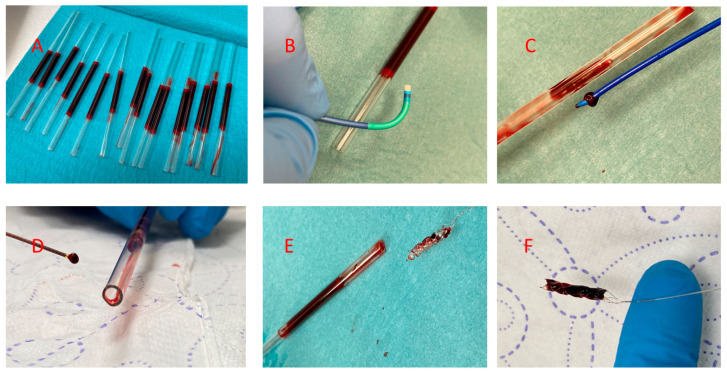
Methodological and practical aspects of the in vitro test. (**A**) An array of 3 mm internal diameter borosilicate glass tubes filled with blood. (**B**) Part of the distal tip of the Launcher catheter and test tube. (**C**) A test tube emptied using a Pronto V4 aspiration catheter with the distal tip obstructed by the clot. (**D**) Part of the test tube and Vasco 21+D catheter with the distal tip obstructed by a clot. (**E**) CatchView stentriever removed from a test tube, where the device has only minimal clot debris in the cells. (**F**) Stentriever completely filled with a clot.

The assessment of the effectiveness of aspiration using the catheters was compared by measuring the weight of human blood clots formed in two different timeframes. Samples of blood were taken from a healthy volunteer (KP) who was not undergoing any treatment for chronic disease. We did not test for factor V Leyden mutation. Collected blood was inserted (0.6 mL) into glass tubes with a 3 mm internal diameter. These blood samples were allowed to clot at room temperature for six and twenty-four hours, respectively. For each thrombus aspiration/removal tool and timeframe, an array of 20 tubes was prepared. In this way, 160 samples were examined. Subsequently, a single-pass aspiration procedure with one of the examined catheters was performed. During these procedures, the thrombectomy device was connected to the aspiration system (syringe), and the tip was placed in direct contact with the thrombus by visual estimation. All these procedures were performed by a single experienced interventional cardiologist (AT) to minimize the impact of the operator’s manual skills on the final results. Figure 1, below, shows the methodological and practical aspects of the experiment.

All the tubes were weighed before and after the aspiration procedure (KERN PCB 100-3 laboratory scale). The mass of the aspirated clot was expressed as the percentage of the initial clot bulk.

### 2.3. Statistics

The initial analysis of the obtained data demonstrated that some groups had an abnormal distribution (Shapiro–Wilk test), and the inhomogeneity of variance was observed. Thus, for a detailed analysis, we used Kruskal ANOVA and Mann–Whitney U tests. Statistically significant differences between the analyzed variables were assumed at *p* < 0.5. Statistica v.13.3, licensed for use by the Medical University of Silesia, was used for all the computations [21].

## 3. Results

### 3.1. Aspiration of 6-Hour Clots

The results are presented in the Table 2 below.

The four examined catheters performed differently in the extraction of the 6 h clots (*p* < 0.001). However, the individual comparisons showed that the CatchView stentriever was significantly less effective when compared to the Vasco+, Pronto V4 and Launcher aspiration catheters (*p* < 0.001 for each of the comparisons). The latter three aspiration devices were able to remove percentages of the thrombus mass approximately 3.5–4.1 times higher than CatchView. Vasco+, Pronto V4 and Launcher were comparably effective in the 6 h clot removal, though Pronto V4 removed the highest percentage of the thrombus mass, while Vasco+ removed the lowest percentage. The Pronto V4 catheter removed 9.1% more of the thrombus mass than the Vasco+ catheter.

### 3.2. Aspiration of 24-Hour Clots

The results are presented in Table 3, below.

The thrombus removal tools examined in our study performed differently for the 24 h clots (*p* < 0.001). The individual comparisons showed that the CatchView stentriever was significantly less effective when compared to the Vasco+, Pronto V4 and Launcher aspiration catheters (*p* < 0.001 for each of the comparisons). The latter three aspiration devices were able to remove percentages of the thrombus mass approximately 3.7–4.5 times higher than CatchView. Vasco+ and Launcher were comparably effective in the 24 h clot removal. The Pronto V4 catheter outperformed all the other devices in the removal of the 24 h clots and was able to remove 14.5% more of the thrombus mass than the Vasco+ catheter.

### 3.3. The Interaction between the Thrombus Aspiration Tools and Clotting Time

**Figure 2:**
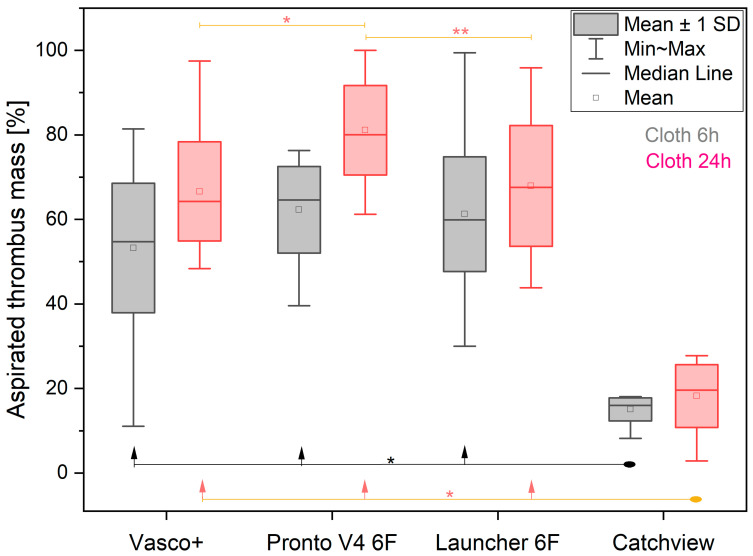
The percentage of the aspirated thrombus mass removed by the different catheters for the 6 h clots (grey) and 24 h clots (red). Significant differences are denoted as * *p* < 0.001, ** *p* < 0.01.

All the thrombus removal tools were more efficient in the extraction of the 24 h clots when compared to the extraction of the 6 h clots (Figure 2). The average percentage of the 6 h clot mass removed by the four pooled catheters was 48.0 ± 22.5%, and the average percentage of the 24 h clot mass removed by the four pooled catheters was 58.5 ± 26.5% (*p* < 0.001). The slightest, yet significant, difference was observed in the performance of CatchView (improvement of 2.9%). The greatest difference was observed in the performance of Pronto V4 (improvement of 19.5%). The Launcher and Vasco+ catheters improved by 6.6% and 13.4%, respectively.

## 4. Discussion

In our in vitro experiment, we showed that the Pronto V4 aspiration catheter was most efficient in performing clot aspiration, while the stent-retriever CatchView Mini 20 was largely ineffective in clot removal.

Stentriever tools are designed for the recanalization of cerebral arteries in patients with acute ischemic stroke [22]. Velioglu et al. recently reported their initial experience of the use of the CatchView stentriever in patients with acute stroke [23]. The use of stentrievers in patients with myocardial infarction has been described in two case reports [18,24]. Uribe et al. described the removal of the thrombus from the left main coronary artery without an occlusive atherosclerotic lesion at the site [18], while Bhoopalan et al. described the removal of the thrombus from an atherosclerotic and ectatic right coronary artery [24]. To explain the inferior performance of CatchView in our experiment, we must consider the difference between the pathomechanisms of acute ischemic stroke and acute myocardial infarction. Coronary artery occlusion is caused by local thrombus formation, while acute ischemic stroke most often occurs due to cerebral vessel embolism. Our in vitro model better represents the local thrombus formation, and these findings may better explain the improved efficacy of the aspiration catheters compared to Catchview. Indeed, stentriever thrombectomy devices have achieved higher recanalization rates, with improved clinical outcomes compared to aspiration catheters [25].

In the present study, Launcher, Vasco and Pronto were comparably effective in the 6 h clot removal. The aforementioned observations indicate that the Launcher guide catheter can be successfully used to remove the thrombus without any additional aspiration catheter required. This could be particularly effective in cases when the thrombus mass is located in the initial areas of the main coronary arteries, such as the beginning of the right coronary artery or before the left main coronary artery bifurcation, where the guide catheter can easily be delivered [13,14,15,16,17,26]. In contrast, microcatheters may be useful in the removal of distal emboli [27].

Moreover, the present study showed that thrombus aspiration efficacy increases with the time allowed for blood clotting. This relationship was mentioned in a study by Chao et al. [28]. The authors concluded that the thrombus removal intervention is time-dependent and only offers a significant benefit when it is performed within 4–8 h after the onset of symptoms. The European Society of Cardiology guidelines indicate that reperfusion therapy is suitable for all patients with symptoms of ischemia of a <12 h duration. Additionally, the primary PCI strategy should be followed for patients with symptoms lasting >12 h in the presence of the following: ECG dynamic changes, ECG evidence of ongoing ischemia, ongoing or recurrent pain and symptoms such as signs of heart failure, shock or malignant arrhythmias [7]. A possible explanation for this time-dependent aspiration efficacy is that, in the early period after the onset of STEMI, the thrombus is relatively soft, being easily fractured and crushed by balloon inflations and, additionally, easily dissolved by thrombolytic drugs. Therefore, mechanical thrombectomy devices may not be greatly effective during early reperfusion therapy in the course of STEMI. As the ischemic time elapses, the thrombus becomes more organized due to continuing fibrin filament polymerization and clot retraction, rendering it more difficult to crush or dissolve. Thus, the clinical impact of thrombus aspiration after a long period following the onset of STEMI symptoms (>12 h) may be considered more efficient. Chao et al. suggested that it is difficult to aspirate old, organized thrombi (>8 h) in the late stage of the course of STEMI. Additionally, they claimed that at this late stage, there is minimal or no residual myocardium to be salvaged, which could suggest that the clinical benefit of thrombus aspiration performed more than 8 h after symptom onset may be negligible [28]. However, other studies have shown that an invasive strategy reduces the myocardial infarct size and improves survival among patients with acute STEMI presenting more than 12 h after symptom onset [29]. Recently, Roberto et al. demonstrated that in-hospital mortality among latecomers (12–48 h after symptom onset) was reduced, and this was possibly associated with the increasing implementation of PCI [30]. Our results might suggest that during the application of an invasive strategy to this group of patients, the greatest benefit can be obtained by using aspiration thrombectomy devices. According to the ESC guidelines, the strategy of routine primary PCI should be considered for patients presenting late (12–48 h) after symptom onset [7].

### Limitations of the Study

The technique of whole-blood clotting in glass tubes used in our investigation and other studies [11,20,31] is not a perfect model of in vivo thrombus formation. The clot formed in vitro lacks the platelet component and contains a differently structured fibrin network. Additionally, in vitro clots retract and do not allow for an additional clot to be formed on the outer clot surface [32,33]. The histological examination of ex vivo thrombi aspirated from culprit lesions has provided information on the significant variability in their content and structure [34,35,36,37]. Therefore, the results of in vitro testing cannot be transferred directly to clinical practice. Our results only indicate the most efficient of thrombus removal devices and facilitate the interventionalist selecting the most appropriate device in the specific clinical setting. Other practical information could be obtained through the testing of the thrombus removal tools by several operators. Thus, we could assess the inter-operator variability. Finally, the development of an ex vivo model of coronary thrombosis or the adaptation of a Badimon chamber would provide the essential results required for a comparison of the aspiration catheters [38]. Thus far, to the best of our knowledge, no in vivo study of thrombus aspiration has utilized in vitro tests to identify the most efficient aspiration catheter for future experiments.

## 5. Conclusions

Large-bore aspiration catheters were found to be more effective than narrow-bore catheters or stent-retrievers in an in vitro model of thrombus removal. The thrombus aspiration efficacy increases with longer thrombus formation times.

## Figures and Tables

**Table 1:** Specifications of the tested devices.

	Vasco+ 21D	Pronto V4	Launcher	CatchV Mini 20
Outer diameter	0.8 mm	1.6 mm	2.0 mm	4.0 mm
The shape of the aspiration lumen	circle 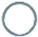	crescent 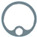	circle 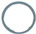	stent-retriever 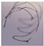
Inner diameter	0.61 mm	1.3 × 0.81 mm	1.8 mm	-
Aspiration lumen area distal	0.29 mm^2^	1.0 mm^2^	2.54 mm^2^	-
Complete information available at	www.balt.fr (accessed on 16 August 2022)	www.teleflex.com (accessed on 16 August 2022)	www.medtronic.com (accessed on 16 August 2022)	www.balt.fr (accessed on 16 August 2022)

**Table 2:**
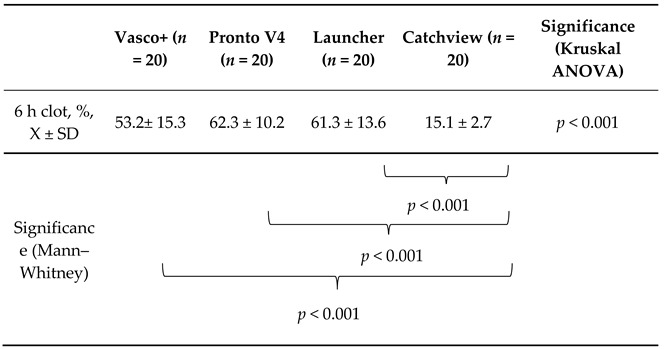
Comparison of the percentages of the aspirated thrombus mass for the 6 h clots. Data presented as mean ± standard deviation.

**Table 3:**
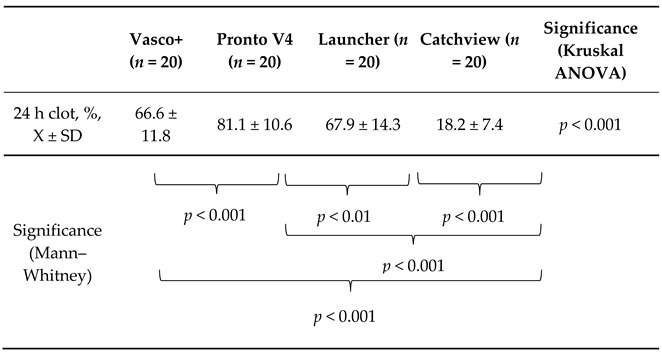
Comparison of the percentages of the aspirated thrombus mass for the 24 h clots. Data presented are mean ± standard deviation.

## Data Availability

The data presented in this study are available on request from the corresponding author.

## Abbreviations

ANOVA	analysis of variance
ECG	electrocardiography
PCI	percutaneous coronary intervention
STEMI-ST	segment elevation myocardial infarction
TIMI	thrombolysis in myocardial infarction
